# Penetrating Cardiac Injury: A 20-Year Retrospective Analysis at a High-Complexity University Center

**DOI:** 10.21470/1678-9741-2024-0049

**Published:** 2025-03-13

**Authors:** Li Siyuan Wada, Paulo Roberto Barbosa Évora, Giovane Okarenski, Adilson Scorzoni Filho, Mauricio Godinho, Sandro Scarpelini, Gabriel Bianco Giuliani, Danilo Tadao Wada, Fabio Luis-Silva, Alfredo José Rodrigues

**Affiliations:** 1 Department of Surgery and Anatomy, Division of Cardiovascular and Thoracic Surgery, Faculdade de Medicina de Ribeirão Preto, Universidade de São Paulo, Ribeirão Preto, São Paulo, Brazil; 2 Department of Surgery and Anatomy, Division of Trauma Surgery, Faculdade de Medicina de Ribeirão Preto, Universidade de São Paulo, Ribeirão Preto, São Paulo, Brazil; 3 Department of Medical Imaging, Hematology, and Clinical Oncology, Thoracic and Cardiovascular Imaging, Faculdade de Medicina de Ribeirão Preto, Universidade de São Paulo, Ribeirão Preto, São Paulo, Brazil; 4 Department of Clinical Medicine, Faculdade de Medicina do Centro, Universitário Barão de Mauá, Ribeirão Preto, São Paulo, Brazil

**Keywords:** Blood Pressure, Cause of Death, Consciousness, Emergency Medical Services, Hemodynamics, Hemorrhage, Hospitals, Medical Records, Mortality

## Abstract

**Introduction:**

Penetrating cardiac injury, though infrequent, is associated with substantial mortality. In 2005, our research team conducted a comprehensive retrospective analysis of penetrating cardiac injuries managed at our facility from 1990 to 2003. Now, two decades later, we conducted the present study on penetrating cardiac injuries attended in our hospital over the last 20 years.

**Methods:**

This is a retrospective analysis of medical records and trauma database data, with a focus on survivors of penetrating cardiac trauma, excluding those deceased upon arrival.

**Results:**

Out of 1,093 cases, 25 had penetrating cardiac injuries with an overall mortality rate of 36%. Hemorrhage was the leading cause of death, and survival was correlated with higher systolic blood pressure upon admission and the level of consciousness.

**Conclusion:**

The study highlights the need for rapid intervention and emphasizes the importance of managing bleeding and supporting hemodynamics. It also points to areas for improvement in emergency care and the benefits of interdisciplinary collaboration.

## INTRODUCTION

Penetrating cardiac injury, though infrequent, is associated with substantial mortality. The historical documentation of cardiac injuries extends back to antiquity, epitomized by the classical account of Sarpedon’s demise^[1]^. The Unidade de Emergência do Hospital das Clínicas (UEHC), which functions as the sole provider of quaternary-level emergency care within the extensive Ribeirão Preto macro-region, serves an expansive community encompassing 34 municipalities with a combined population of 1.75 million. This institution is an affiliate of the Faculdade de Medicina de Ribeirão Preto at the Universidade de São Paulo.

In 2005, our research team conducted a comprehensive retrospective analysis, compiling incidents of penetrating cardiac injuries managed at our facility from 1990 to 2003. Now, two decades after our initial study, the present research endeavors to provide an updated examination of the trends and patient outcomes associated with the management of this intricate and distinct form of trauma.

The purpose of this investigation is to assess the demographic characteristics, clinical interventions, and prognoses of individuals with penetrating cardiac injuries managed at a tertiary trauma center. The intents are to furnish a contemporary perspective and to elucidate the progression of therapeutic approaches spanning the past two decades.

## METHODS

The present study received approval from the Research Ethics Committee of our institution, as evidenced by Ethical Appreciation Presentation certificate number 66446322.8.0000.5440, with approval granted on January 10, 2023 (opinion number 5.847.195). Consequent to this approval and pursuant to CNS Resolution 466/2012, the research project and the request for a waiver of the Informed Consent Form were both sanctioned.

Our research entailed an observational, retrospective analysis utilizing patient data from the medical records and trauma database of the UEHC. The cohort comprised all survivors of penetrating cardiac trauma treated between January 1, 2003, and December 31, 2022, explicitly excluding individuals who were deceased on arrival (as determined by the absence of cardiac rhythm, arterial pressure, or palpable central pulses).

In 2014, the UEHC transitioned from paper to electronic medical records. Coinciding with this transition was the establishment of the Trauma Database (TDB), supported by a specialized team tasked with compiling data from all cases managed in the Emergency Department of the UEHC. We scrutinized records for all admissions to the UEHC with International Classification of Diseases 10^th^ codes pertinent to thoracic trauma from January 1, 2003, to December 31, 2013, archived in the Medical Archive Service (MAS). Data from 2014 onward were sourced directly from the TDB.

Data collected included hospital record number, date of admission, age at admission, sex, trauma mechanism, injury location as ascertained by physical examination, initial clinical indicators (systolic blood pressure [SBP], heart rate, level of consciousness, presence of jugular venous distension, muffled heart sounds, presence of hemothorax), intraoperative findings (involving cardiac chambers, major vessels, lungs, abdominal organs), date and cause of death, and duration of hospital stay. Patients with a Glasgow Coma Scale score < 8 were classified as unconscious. Patients with inaudible SBP and unconscious were deemed to be in extremis. We excluded cases where the injury did not penetrate beyond the pericardium, that is, all included cases have at least a myocardial injury. We deliberately restricted our study to only those cases confirmed through surgical intervention, with cardiac injury unequivocally stated in the surgical dossier. Notably, despite the sophisticated infrastructure, there is no dedicated thoracic and cardiac surgical team present on site, and none of the surgeries employed extracorporeal circulation. All surgical interventions are executed by the trauma surgery team. The selection of surgical techniques employed in each case was subjected to the individual surgeon's discretion

The primary endpoint was mortality. Nominal qualitative dependent variables with < 5 occurrences in the contingency table were evaluated using Fisher’s exact test, while those with ≥ 5 were assessed using the Chi-Square test. Quantitative dependent variables were tested for normality using the Shapiro-Wilk test; those with normal distribution were analyzed using the independent *t*-test, and those without it were assessed with the Mann-Whitney U test. Data analyses were performed using IBM Corp. Released 2019, IBM SPSS Statistics for Mac OS, version 26.0, Armonk, NY: IBM Corp., with a *P*-value < 0.05 considered statistically significant.

## RESULTS

Utilizing the MAS and the TDB, we identified 1,093 cases of penetrating thoracic trauma managed at the UEHC from January 1, 2003, to December 31, 2022. After meticulous record examination and application of exclusion criteria, 25 cases of penetrating cardiac injury, admitted alive to the UEHC, were ascertained. The cohort consisted of 17 patients (68.0%) with stab wounds (SW) and eight (32.0%) with gunshot wounds (GSW), with none exhibiting evident head trauma. The demographic profile revealed 23 male patients (92.0%) and two females (8.0%), with a mean age of 36 ± 10 years. Of these, nine patients succumbed to their injuries. GSW were implicated in three (33.3%) of these fatalities, while SW were responsible for the remaining six (66.7%), with hemorrhage being the leading cause of mortality (88.9%). There was no significant statistical difference in age and sex between those who died and those who survived. The mean hospital stay was markedly shorter for the deceased (1 ± 4 days) compared to the survivors (26 ± 25 days, t[23]=2.894; *P*=0.008)). Upon admission, the average SBP was 79 ± 45 mmHg, with survivors presenting a higher average SBP (99 ± 31 mmHg) than the deceased (45 ± 48 mmHg; *P*=0.011). The initial mean heart rate was 100 ± 30 bpm, with a slightly higher rate in survivors (104 ± 22 bpm) compared to the deceased (93 ± 42 bpm; t[23]=0.930; *P*=.362). Fifteen patients (60.0%) were conscious upon admission, and 10 patients (40.0%) were unconscious (*P*=.041). Jugular venous distension was observed in eight patients (34.8%), and muffled heart sounds in 11 (50.0%). Beck's triad was present in only four patients (17.4%), and two were in extremis (8.0%). Hemothorax was identified in 24 cases (88.0%) (Table 1). On physical examination upon admission, 20 patients had injuries in the left anterior hemithorax (80.0%), four in the thoracoabdominal transition (16.0%), three in the right posterior hemithorax (12.0%), two in the right lateral thoracic wall (8.0%), one in the right anterior hemithorax (4.0%), one in the left posterior hemithorax (4.0%), and one in the left lateral thoracic wall (4.0%). Three patients (12.0%) underwent emergency department thoracotomy (EDT). Nine patients (36.0%) were approached through left anterolateral thoracotomy, five (20.0%) through left lateral thoracotomy, five (20.0%) through sternotomy, five (20.0%) through bithoracotomy (Clamshell), and one (4.0%) through right anterolateral thoracotomy. Laparotomies were concurrently performed in four individuals (16.0%) (Table 2). None of those 25 cases was treated solely with a pericardial window.

**Table 1:** Demographic data and clinical data.

	All n = 25	Survivors n = 16	Non-survivors n = 9	*P*-value
Age	36 ± 10	36 ± 11	36 ± 9	0.649
Sex				0.145
Male	23 (92.0%)	15 (93.8%)	8 (88.9%)	
Female	2 (8.0%)	1 (6.3%)	1 (11.1%)	
Mechanism of injury				1.000
Gunshot wounds	8 (32.0%)	5 (31.3%)	3 (33.3%)	
Stab wounds	17 (68.0%)	11 (68.8%)	6 (66.7%)	
Signals and symptoms				
Cardiac rate (bpm)	100 ± 30	104 ± 22	93 ± 42	0.589
SBP (mmHg)	79 ± 45	99 ± 31	45 ± 48	0.011
Unconscious	10 (40.0%)	4 (25.0%)	6 (66.7%)	0.041
In extremis	2 (8.0%)	0 (0.0%)	2 (22.2%)	0.120
Jugular venous distention	8 (34.8%)	5 (33.3%)	3 (37.5%)	0.842
Beck’s triad	4 (17.4%)	3 (20.0%)	1 (12.5%)	1.000
Hemothorax	22 (88.0%)	13 (81.3%)	9 (100.0%)	0.166
EDT	3 (12.0%)	0 (0.0%)	3 (33.3%)	0.037
Hospital length of stay (days)	17 ± 23	26 ± 25	1 ± 4	0.008

EDT=emergency department thoracotomy; SBP=systolic blood pressure

**Table 2:** Sites of injury and surgical incisions.

	All n = 25	Survivors n = 16	Non-survivors n = 9	*P*-value
Site of injury				
Left anterior chest	20 (80%)	12 (75.0%)	8 (88.9%)	0.405
Right anterior chest	1 (4%)	1 (6.3%)	0 (0.0%)	1.000
Left posterior chest	1 (4%)	0 (0.0%)	1 (11.1%)	0.360
Right posterior chest	3 (12%)	3 (18.8%)	0 (0.0%)	0.280
Left lateral chest	1 (4%)	1 (6.3%)	0 (0.0%)	1.000
Right lateral chest	2 (8%)	2 (12.5%)	0 (0.0%)	0.520
Thorax-abdomen transition	4 (16%)	3 (18.8%)	1 (11.1%)	1.000
Surgical incision				0.525
Left lateral thoracotomy	5 (20%)	4 (25.0%)	1 (11.1%)	
Left anterior-lateral thoracotomy	9 (36%)	4 (25.0%)	5 (55.6%)	
Sternotomy	5 (20%)	4 (25.0%)	1 (11.1%)	
Clamshell	5 (20%)	3 (18.8%)	2 (22.2%)	
Right anterior-lateral thoracotomy	1 (4%)	1 (6.3%)	0 (0.0%)	

The cardiac chambers most frequently affected were the left ventricle (LV) and right ventricle (RV), each in 32.0% of cases, followed by the left atrium and right atrium, each in 12.0%, with the LV being predominantly affected in the deceased group (55.6%) and the RV in the survivor group (43.8%) (Figure 4). Multiple chamber involvement occurred in two patients (8.0%). Other intrathoracic structures affected were the lung (36.0%), pulmonary artery (8.0%), vena cava (8.0%), pulmonary veins (8.0%), and coronary artery (4.0%). Three patients (12.0%) had concomitant abdominal injuries, with the liver being the most frequently involved organ (8.0%), followed by the stomach, small intestine, and colon (each 4.0%) (Table 3).

**Table 3:** Wounded chambers and associated injuries.

Injuries	All n = 25	Survivors n = 16	Non-survivors n = 9	*P*-value
Thorax				
Right atrium	3 (12.0%)	1 (6.3%)	2 (22.2%)	0.530
Left atrium	3 (12.0%)	2 (12.5%)	1 (11.1%)	1.000
Right ventricle	8 (32.0%)	7 (43.8%)	1 (11.1%)	0.182
Left ventricle	8 (32.0%)	3 (18.8%)	5 (55.6%)	0.087
Coronary artery	1 (4.0%)	0 (0.0%)	1 (11.1%)	0.360
Ascending aorta	0 (0.0%)	0 (0.0%)	0 (0.0%)	-------
Pulmonary artery	2 (8.0%)	1 (6.3%)	1 (11.1%)	1.000
Venae cavae	2 (8.0%)	1 (6.3%)	1 (11.1%)	1.000
Pulmonary veins	2 (8.0%)	2 (12.5%)	0 (0.0%)	0.520
Lungs	9 (36.0%)	5 (31.3%)	4 (44.4%)	0.671
Abdomen				
Liver	2 (8.0%)	1 (6.3%)	1 (11.1%)	1.000
Stomach	1 (4.0%)	1 (6.3%)	0 (0.0%)	1.000
Spleen	0 (0.0%)	0 (0.0%)	0 (0.0%)	-------
Small bowel	1 (4.0%)	0 (0.0%)	1 (11.1%)	0.360
Colon	1 (4.0%)	0 (0.0%)	1 (11.1%)	0.360

## DISCUSSION

Our data indicated a greater prevalence of SW at 68.0%, compared to GSW at 32.0%, a distribution that might be reflective of underlying regional socioeconomic factors. Some studies show that SW is more frequent than GSW^[2,3,4,5,6]^, while others show that GSW and SW have a similar incidence^[7,8,9,10,11]^. As in the literature, the vast majority of our sample was male (92.0%), with an average age between the third and fourth decade of life (average age 36 ± 10 years)^[1,2,3,4,5,6,7,8,9,10,11,12]^.

The mortality rates for penetrating cardiac trauma reported in the literature are highly variable, ranging from 14.2%^[13]^ to 94.0%^[2]^. In our current study, the overall mortality was 36.0%, higher than our previous study from 2005 (14.2%)^[13]^. This rise may be attributable to advancements in pre-hospital care, allowing more critically injured patients to reach the hospital alive.

Exsanguination remained the leading cause of death, accounting for 88.9% of fatalities, underscoring the lethality of penetrating cardiac injuries, particularly within the first 24 hours post-admission. This finding was consistent with our previous research (82.6%).

**Fig. 1:**
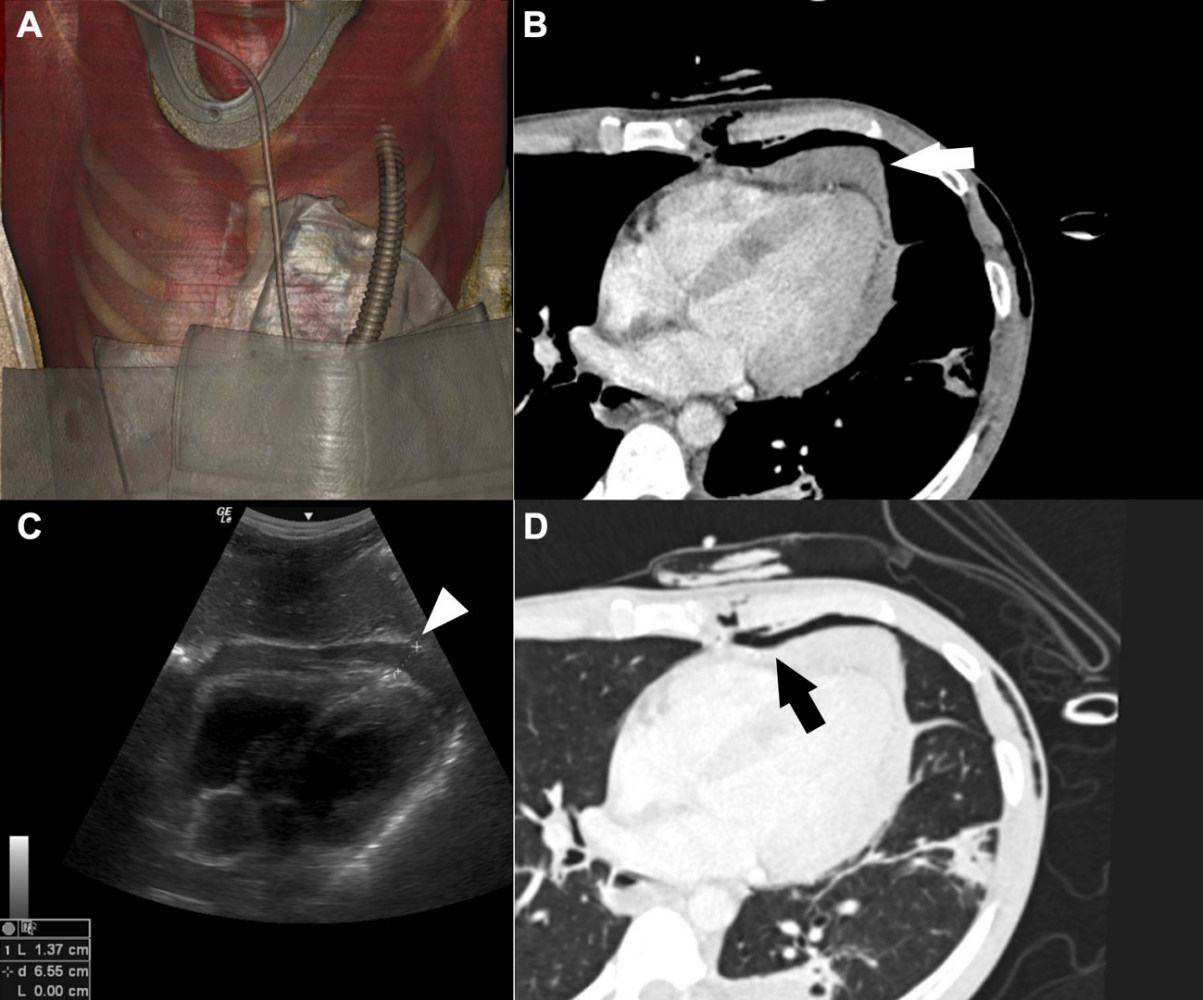
Computed tomography reconstruction with volume rendering (A) illustrating a compressive dressing on a patient with a stab wound in the precordial region. The exam identified the presence of hemopericardium (white arrow and white arrowhead) in the mediastinal window (B), which had already been identified by FAST (C). Note the wound tract in the thoracic wall with the presence of pneumothorax (black arrow) in the lung window (D).

EDT, performed in 12.0% of our patients, was a significant variable differentiating survivors from non-survivors, indicative of its use in critically ill patients. In the prospective study by Mina et al.^[8]^, in 2017, the mortality of those who underwent emergency thoracotomy was 86.0%, while those who underwent thoracotomy in the surgical center was 24.0%, being considered a prognostic factor in penetrating cardiac injuries. Tyburski et al.^[11]^, in 2000, also showed that emergency thoracotomy is associated with a lower survival rate (*P*<.001). In the prospective study by Asensio et al.^[10]^ from 1998 about predictors of outcome in patients with penetrating cardiac injury, patients undergoing emergency thoracotomy in the trauma room had a mortality of 84.0% and it was considered a predictor of unfavorable outcome (*P*<.0001) (Figure 1).

**Fig. 2:**
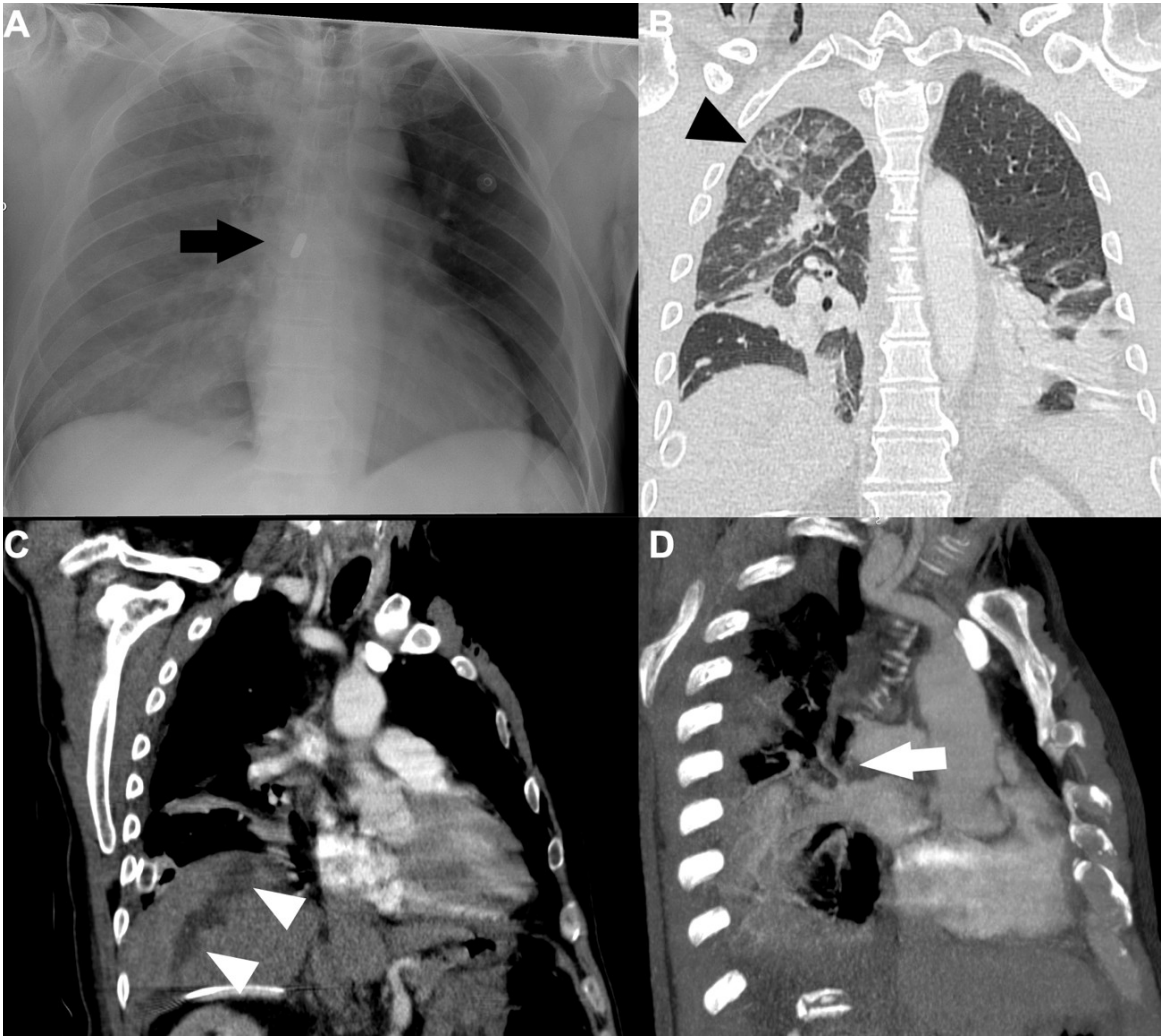
X-ray image (A) of a patient with a gunshot wound with the bullet lodged inside the left atrium (black arrow). The patient underwent surgical approach with bullet removal and suture of the superior right pulmonary vein. In the control tomographic exam after the surgery (B, C, and D), it is possible to identify signs of localized edema in the upper lobe of the right lung (black arrowhead), the trajectory taken by the projectile from the right hypochondrium towards the heart (white arrowheads), in addition to the suture region of the superior right pulmonary vein (white arrow).

The average SBP upon admission in the survivor group (99 ± 31 mmHg) is higher than in the deceased group (45 ± 48 mmHg; *P*=.011). The studies by Mina^[8]^, Fraga^[9]^, and our group^[13]^ also demonstrated the same result. Tyburski's multivariate analysis also highlighted admission SBP as a critical prognostic indicator^[11]^. Fifteen patients (60.0%) were conscious on admission, and 10 patients (40.0%) were unconscious (*P*=.046), reaffirming the association between loss of consciousness and increased mortality, as reported in our 2005 publication and by other authors (*P*=.003)^[13]^. The same result was found in the works of Mina^[8]^, Fraga^[9]^, and Asensio^[10]^. Since none of the 25 patients had injury to the head and neck region, unconsciousness was attributed to cerebral hypoperfusion, likely due to severe blood loss. Unconscious patients were probably more severe for having lost more blood volume, as suggested by Asensio^[14]^ (Figure 2).

**Fig. 3:**
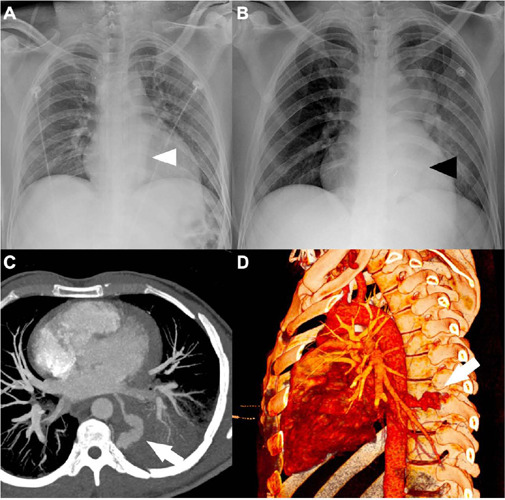
Patient with a stab wound in the dorsal paravertebral region on the left. The entry radiography (A) showed a retrocardiac opacity (white arrowhead), which increased in a few hours (B). In the computed tomography scan (C and D) of the patient, it was possible to identify an injury in the posterior wall of the left atrium with active bleeding (white arrows).

As there are few reports in the surgical records about the presence of cardiac tamponade, this was not included in the analysis. However, jugular venous distension was noted in 34.8% of patients and muffled heart sounds in 50.0%. Beck's triad was present in only four patients (17.4%). The infrequency of these classical signs might be explained by concurrent hypovolemia, a hypothesis supported by the works of Asensio^[10]^ and our previous studies^[13]^. Over twenty years, a predominance of SW has been observed in comparison to GSW, potentially reflecting the socioeconomic particularities of the Ribeirão Preto region. The observed mortality rate of 36.0% represents an increase from prior studies, possibly reflecting enhancements in pre-hospital care and the ability of emergency services to sustain patients with more severe injuries who might not have previously reached hospital care (Figure 3).

**Fig. 4:**
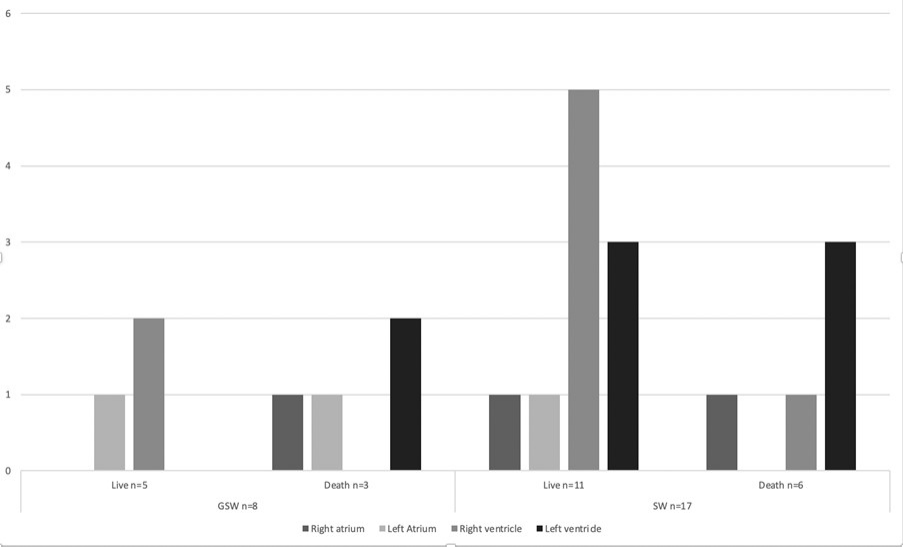
Distribution according to mechanism of injury, localizations of wounds, and mortality. GSW=gunshot wounds; SW=stab wounds.

Key prognostic indicators such as SBP at admission and the level of consciousness on arrival have been substantiated as significant, aligning with the literature and underscoring the criticality of prompt and efficacious emergency interventions. The preponderance of mortality due to exsanguination calls for continued emphasis on aggressive bleeding control and hemodynamic stabilization strategies.

Furthermore, this study illuminates the progression in managing penetrating cardiac injury at a quaternary trauma center, with implications for clinical practice and health policy. Despite advances, the inherent limitations of emergency care, including the lack of an in-house thoracic and cardiac surgical team, denote areas ripe for enhancement.

### Limitations

This study has several limitations. Firstly, it is a retrospective observational study based on the database of a single institution. The second limitation is that, as it involves rare cases with a high pre-hospital mortality rate, the sample size is small. This fact is also observed in the literature review articles. In the bibliographic review, the vast majority of the works found consist of case reports, and among the few review articles and systematic reviews, the casuistry is relatively small.

## CONCLUSION

In summation, our findings underscore the imperative of maintaining alertness and modifying trauma care approaches to augment patient outcomes following penetrating cardiac injuries. It is essential to foster ongoing interdisciplinary collaboration among emergency medical services, trauma care, and cardiovascular surgery to refine protocols that can further diminish the mortality and morbidity rates of these severe conditions.

## Data Availability

The datasets generated and/or analyzed during the current study are available from the corresponding author on reasonable request. Requests for access to these datasets should be directed to Li Siyuan Wada, with permission of Research Ethics Committee of Hospital das Clínicas of Faculdade de Medicina de Ribeirão Preto, Universidade de São Paulo.
